# Supplementary material for: Gradients of connectivity distance in the cerebral cortex of the macaque monkey
Source: Brain Struct Funct. 2018 Dec 13;224(2):925–35. doi: 10.1007/s00429-018-1811-1 (PMC6420469; doi:10.1007/s00429-018-1811-1)
Supplement: Supplementary file 1 — Supplementary material 1 (DOCX 1161 KB) [file 429_2018_1811_MOESM1_ESM.docx]

# Online Resource

Brain Structure and Function

# Gradients of connectivity distance in the cerebral cortex of the macaque monkey

Sabine Oligschläger, Ting Xu, Blazej M. Baczkowski, Marcel Falkiewicz, Arnaud Falchier, Gary Linn, Daniel S. Margulies^*^

* Correspondence:

Institut du Cerveau et de la Moelle épinière

Hôpital Pitié-Salpêtrière

47, boulevard de l’Hôpital

+33 (0) 1 57 27 41 37

daniel.margulies@icm-institute.org

### Online Resource Figure 1

###
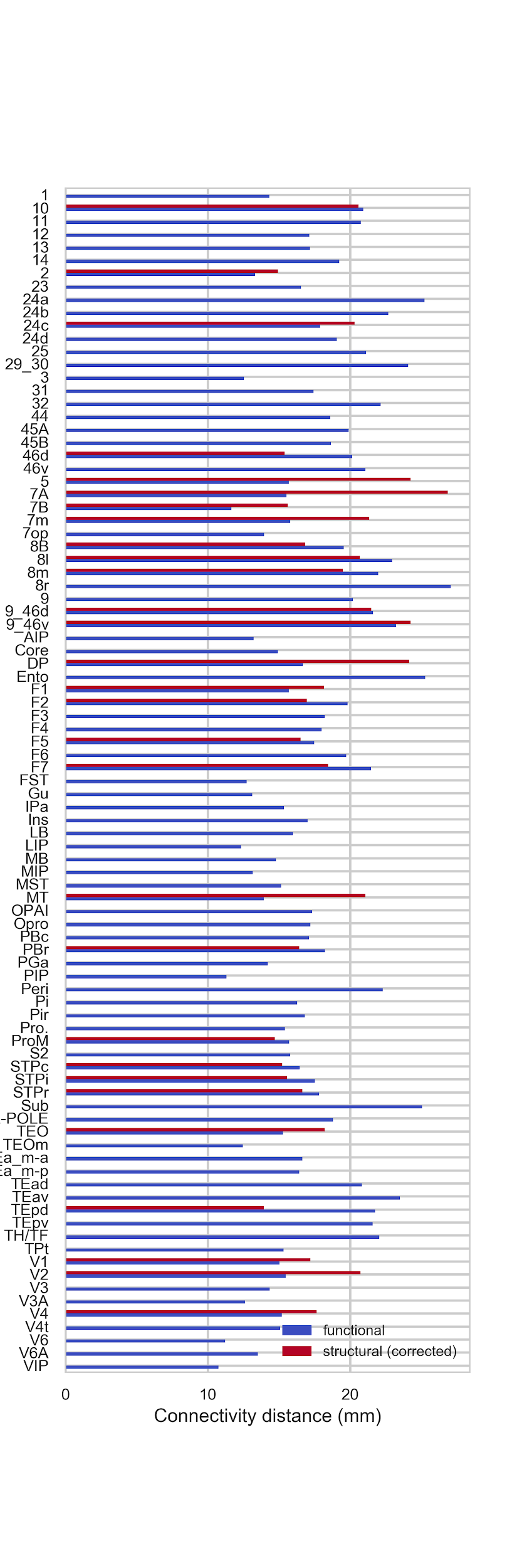


###

###

###

###

###

###

###

###

###

###

###

###

Functional (blue) and structural (red) connectivity distance for each area included in the analysis.

### Online Resource Figure 2


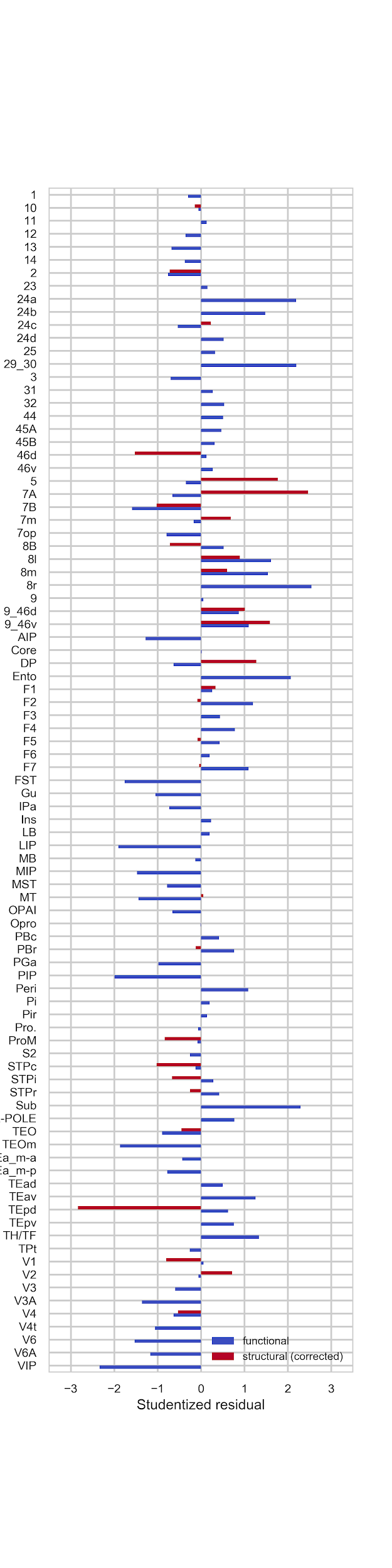


###

###

###

###

###

###

###

###

###

###

###

###

###

### Studentized residuals for each area included in the general linear models (GLMs) that model functional (blue) and structural (red) connectivity distance as function of distance from primary sensory-motor cortex.

### Online Resource Figure 3


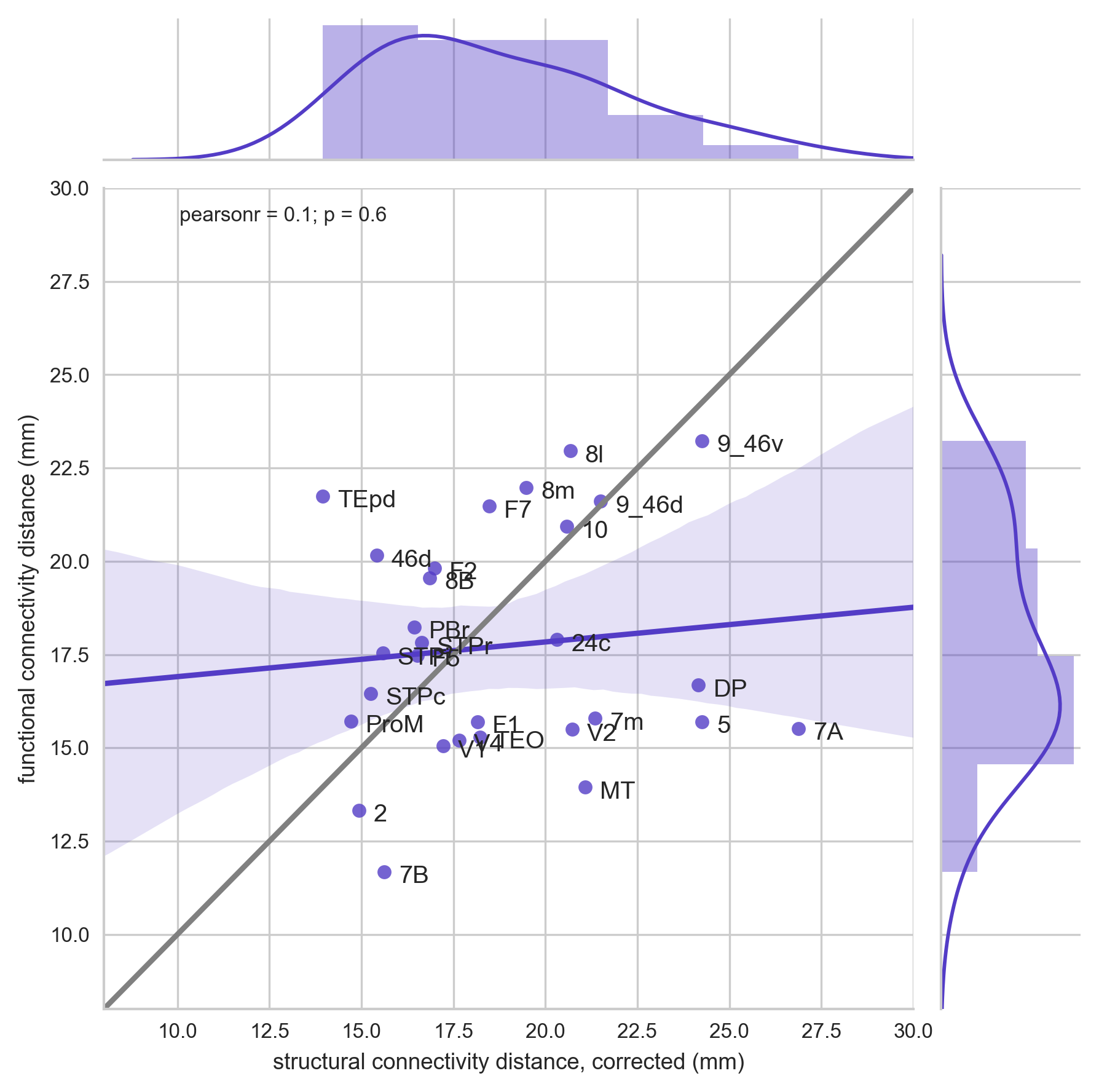


Topographic similarity between functional and structural connectivity distance. For reference, the grey line indicates a perfect correspondence between the two measures.

### Online Resource Figure 4

**
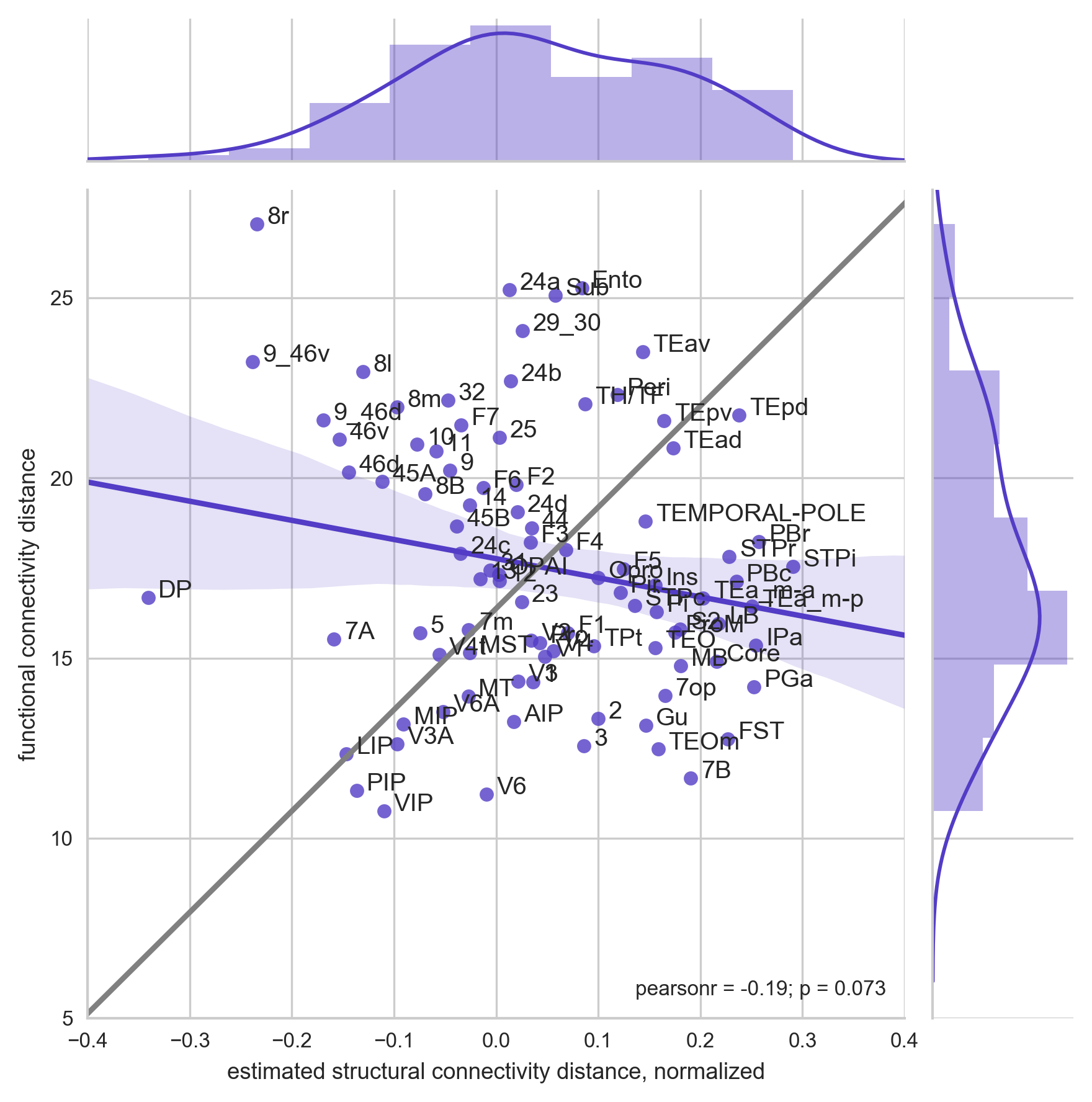
**

Topographic similarity between functional connectivity distance and estimated whole-brain structural connectivity distance. For reference, the grey line indicates a perfect correspondence between the two measures.

### Online Resource Figure 5

**
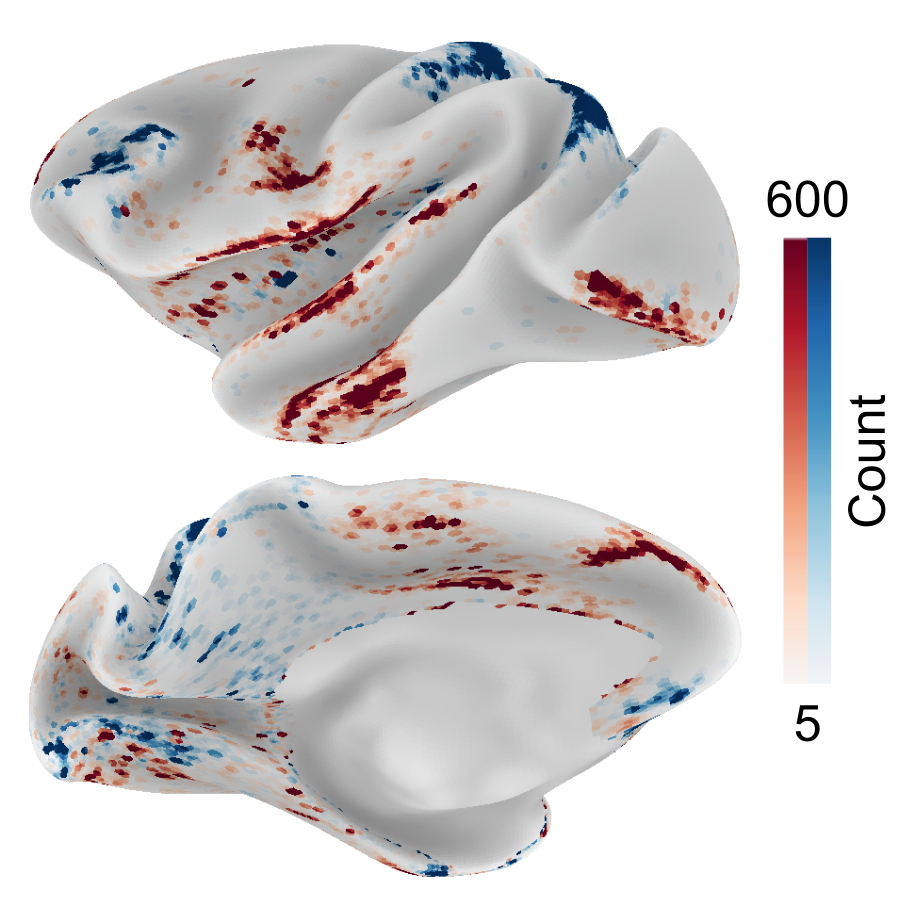
**

Hypothesis-free search for anchor regions: Optimization procedure. To estimate the distribution of structural connectivity distance without the combinatorial problem of random sampling locations on the cortical surface, we implemented a optimization algorithm. Starting with a random set of nodes, these nodes were then moved in steps of 1 mm towards the direction of higher variance explained until a stable set of locations was found. The search was repeated 1000 times. The map counts the times a node was included in the final set and distinguishes between positive (the further from reference location, the longer the connectivity distance) and negative (the further from reference location, the shorter the connectivity distance) relationships.
